# Maßnahmen zur Sicherstellung der ambulanten Versorgung in ländlichen und strukturschwachen Regionen – eine narrative Übersichtsarbeit

**DOI:** 10.1007/s00103-025-04119-0

**Published:** 2025-09-04

**Authors:** Jobst Augustin, Nirohshah Trialonis-Suthakharan, Enno Nowossadeck

**Affiliations:** 1 Institut für Versorgungsforschung in der Dermatologie und bei Pflegeberufen (IVDP), Universitätsklinikum Hamburg-Eppendorf (UKE), Martinistraße 52, 20246 Hamburg, Deutschland; 2 Fachgebiet Soziale Determinanten der Gesundheit, Abt. für Epidemiologie und Gesundheitsmonitoring, Robert Koch-Institut (RKI), Berlin, Deutschland

**Keywords:** Regionen, Versorgungsmodelle, Telemedizin, Bewertung, Evaluation, Space, Care models, Telemedicine, Benefit assessment, Evaluation

## Abstract

Zur Sicherstellung der Versorgung in ländlichen und strukturschwachen Regionen werden verschiedene Public-Health-Maßnahmen eingesetzt. Deren tatsächlicher Effekt ist bislang jedoch nicht vollends geklärt. Ziel dieser narrativen Übersichtsarbeit ist es, entsprechende Maßnahmen zu identifizieren und vorliegende Evaluationsergebnisse zu deren Wirksamkeit zusammenzutragen.

Im Rahmen einer Literatur- und Dokumentenanalyse wurden neben Google Scholar gezielt Webseiten und Datenbanken der Selbstverwaltung (z. B. kassenärztliche Vereinigungen, Ministerien) im Gesundheitswesen sowie einschlägiger deutscher Fachzeitschriften durchsucht. Die Ergebnisse zeigen zahlreiche konzeptionelle Ansätze und praktische Maßnahmen zur Sicherstellung der Versorgung. Diese werden als Einzelmaßnahme (z. B. „Rollende Arztpraxis“) oder komplexes Bündel von Maßnahmen (z. B. „Digitales Dorf“) umgesetzt. Maßnahmen zur Verbesserung der Kontextgestaltung (z. B. Niederlassungsförderung durch günstiges Bauland) und neue Organisationformen (z. B. Ärztenetze) werden häufig angewendet, sind in ihrer Wirkung aber oftmals begrenzt. Aufgrund ihrer relativ einfachen Implementierung in die Praxis haben telemedizinische Maßnahmen eine besondere Bedeutung und werden in ihrem Effekt überwiegend positiv bewertet.

Grundsätzlich sollte die Bewertung von Maßnahmen weiterhin intensiviert und auch den Methoden und Kriterien für eine Bewertung des Effektes verstärkt Beachtung gewidmet werden. Eine Begleitevaluation sollte auch dazu dienen, dass Maßnahmen bei erfolgreicher Evaluierung nach Projektende nicht eingestellt, sondern bei entsprechendem Effekt in die Regelversorgung überführt werden. Aufgrund regionaler und lokaler Gegebenheiten sind zudem regionsspezifische Lösungen zur Sicherstellung und Optimierung der Versorgung anzustreben.

## Abstract (en)

Various public health measures are being implemented to ensure healthcare provision in rural and structurally vulnerable regions. However, the actual effect of these measures has not yet been fully clarified. This narrative review aims to identify relevant measures and compile existing evaluation results on their effectiveness.

As part of a literature and document analysis, Google Scholar was used to search specific websites and databases of self-governing bodies in the healthcare sector (e.g. associations of statutory health insurance physicians and ministries) as well as relevant German journals. The results reveal a variety of conceptual approaches and practical measures designed to ensure healthcare provision. These are implemented as individual measures (e.g. “Rollende Arztpraxis”) or complex bundles of measures (e.g. “Digitales Dorf”). Measures to improve the context, such as promoting the establishment of practices through the provision of affordable building land, and new organisational structures, such as doctor networks, are frequently employed, but their impact is often limited. Due to their relatively simple implementation, telemedicine measures are particularly important and are predominantly rated as having a positive effect.

In principle, the evaluation of measures should be intensified further, with greater attention being paid to the methods and criteria used to evaluate their effectiveness. An accompanying evaluation should also ensure that successfully evaluated measures are not discontinued at the end of the project, but transferred to standard care if they are effective. Due to regional and local conditions, region-specific solutions should also be sought to optimise care.

## Hintergrund

Die Gewährleistung einer flächendeckend ambulanten ärztlichen Versorgung, wie vom Gesetzgeber gefordert (§ 75 Abs. 1 und § 73 Abs. 2 Sozialgesetzbuch (SGB) Fünftes Buch (V) [1]) hat sich in der Umsetzung als schwierig erwiesen. Dies betrifft vor allem die Gesundheitsversorgung in ländlichen und strukturschwachen Räumen, die oftmals noch nicht ausreichend gewährleistet ist [2].

Nach Auschra et al. [3] bestehen 5 wesentliche Ursachen für Unterversorgung: demografischer Wandel, Binnenmigration, Fachkräftemangel, ineffiziente Verteilung von Leistungsanbietern und Konzentration ärztlicher Profession in urbanen Räumen. Der demografische Wandel kann wohl, zumindest mittel- und langfristig, als das zentrale Problem angesehen werden. Dieser führt nicht nur dazu, dass die Bevölkerung älter und damit auch morbider wird, sondern auch das versorgende Personal (Ärzt:innen, Angestellte in Praxen und Pflegeeinrichtungen).

Mit zunehmendem Lebensalter steigen aufgrund der Altersassoziation vieler Krankheitsentitäten die Wahrscheinlichkeit des Auftretens von Krankheiten und damit auch die Nachfrage nach Gesundheitsleistungen bzw. Gesundheitsversorgung. Darüber hinaus führen Abwanderungsprozesse, insbesondere junger Erwerbsfähiger, aus strukturschwachen und ländlichen Regionen in urbane Ballungsräume dazu, dass der Anteil älterer Menschen in diesen Regionen ansteigt, was den Druck auf die Gesundheitsversorgung nochmals erhöht. Daraus resultiert eine zunehmende Diskrepanz zwischen einer wachsenden Nachfrage von Gesundheitsleistungen durch die Bevölkerung und gleichzeitig einem reduzierten Angebot von versorgenden Einrichtungen aufgrund von Praxisschließungen und Fachkräftemangel.

Den Verlierern, den ländlichen und strukturschwachen Räumen, stehen Regionen wie beispielsweise Hamburg als Gewinner gegenüber, die sich durch eine umfangreiche Infrastrukturausstattung, gute Erreichbarkeitsverhältnisse und wirtschaftliche Stärke auszeichnen und insbesondere junge Menschen anziehen. Dies zeigt sich auch in den Wiederbesetzungen von Arztsitzen, denn neben einem Mangel an Mediziner:innen und einer geringen Vergütung von Leistungen in der Grundversorgung [4, 5] passt das Landarztsein oftmals nicht mehr zum Lebensentwurf vieler junger Mediziner:innen [6]. Die meisten der Absolvent:innen zieht es eher in Ballungsräume als in strukturschwache oder ländliche Räume. Der Austausch mit anderen Mediziner:innen, ein vielseitiges Freizeitangebot, sichere Kinderbetreuung, gute Anbindung an öffentlichen Personennahverkehr (ÖPNV), Teilzeitarbeitsmöglichkeiten (Work-Life-Balance) sind nur einige Gründe [7, 8]. Die räumlich ungleiche Verteilung von Leistungserbringern ist ebenfalls eine Ursache von Unterversorgung in strukturschwachen oder ländlichen Regionen und hängt oftmals mit der infrastrukturellen Benachteiligung dieser Räume zusammen (z. B. fehlende Schulen).

Verschiedene gesundheitspolitische Instrumente, allen voran die Bedarfsplanung, sollen die Gesundheitsversorgung in unterversorgten Regionen adjustieren und optimieren. Darüber hinaus existieren mittlerweile zahlreiche Modelle und Maßnahmen, die eine langfristige Sicherstellung der gesundheitlichen Versorgung jenseits der klassischen Bedarfsplanung ermöglichen sollen [9], um damit der existierenden und auch weiterhin drohenden Unterversorgung zu begegnen. Bislang fehlt allerdings ein Überblick zu existierenden Modellen und Maßnahmen und vor allem zu ihrem Effekt auf die Sicherstellung der Gesundheitsversorgung der Bevölkerung in strukturschwachen und ländlichen Regionen. Ziel dieser narrativen Übersicht ist es zu identifizieren, welche Public-Health-Maßnahmen zur Sicherstellung der Versorgung in strukturschwachen und ländlichen Regionen existieren und welche Evaluierungsergebnisse zum Effekt dieser Maßnahmen vorliegen.

Anzumerken ist, dass die in dieser Studie berücksichtigten Maßnahmen vorrangig der Gesundheitsversorgung zuzuordnen sind, angelehnt an die Bundeszentrale für gesundheitliche Aufklärung (BZgA) [10], Nowak et al. [11] und RKI [12], diese jedoch die Gesundheitsversorgung der gesamten Bevölkerung betreffen und daher als Bestandteil von „New Public Health“ verstanden werden. Präventive und gesundheitsfördernde Interventionen, wie zum Beispiel Lotsenmodelle, Gesundheitskioske, Gesundheitsregionen, regionale integrierte Versorgungsmodelle, konnten in der Untersuchung nicht im Detail berücksichtigt werden.

Das grundsätzliche methodische Vorgehen basiert auf einer Literaturrecherche und anschließenden Dokumentenanalyse unter Berücksichtigung von zuvor definierten Suchbegriffen sowie Ein- und Ausschlusskriterien. Die Recherche erfolgte in Google Scholar sowie spezifisch auf den Webseiten aller Gesundheitsministerien auf Bundes- und Landesebene, bei allen kassenärztlichen Vereinigungen (KV) sowie bei weiteren ausgewählten Institutionen (z. B. Optimedis, Gemeinsamer Bundesausschuss). Darüber hinaus wurde eine direkte Suche in den Datenbanken beziehungsweise Archiven einschlägiger deutscher Fachzeitschriften (*Bundesgesundheitsblatt, Das Gesundheitswesen, Deutsches Ärzteblatt *sowie* Zeitschrift für Evidenz, Fortbildung und Qualität im Gesundheitswesen*) vorgenommen. Die Literaturrecherche und Dokumentenanalyse wurden im Oktober und November 2023 durchgeführt und beziehen sich auf Deutschland. Der Fokus lag auf dem ambulanten Bereich, allerdings wurden Ergebnisse aus dem stationären Sektor mit einbezogen, wenn sie von besonderer Bedeutung für die Studie waren.

Alle so gefundenen Publikationen wurden nach ihrem Regionsbezug (allgemein oder ländlich) sowie in Anlehnung an die Gestaltungselemente für Versorgungsmodelle von Auschra et al. [3] charakterisiert. Die Gestaltungselemente nach Auschra et al. werden differenziert nach Maßnahmen der Kontextgestaltung (z. B. Niederlassungsförderung, Öffnung der Krankenhäuser für die ambulante Behandlung), Profession (z. B. Ausbildung und Training von Fachpersonal), Mobilität (z. B. Erhöhung individueller Mobilität von Ärzt:innen und Patient:innen), Technik (z. B. Telemedizin, elektronische Patientenakte), Organisation und Organisationsformen (z. B. Kooperation zwischen regionalen Akteuren, Ärztenetze).

Der für die Studie relevante Teil von Informationen lag primär in Form von Fachzeitschriften vor, darunter vor allem Originalarbeiten sowie Projektberichten, Übersichtsarbeiten und Stellungnahmen. Tab. 1 zeigt Übersicht und Einordnung relevanter Publikationen. Neben dem Regionsbezug werden diese auch nach den Maßnahmen (nach Auschra et al. [3]) charakterisiert. Die Artikel von Geraedts et al. [13] sowie von Schubert et al. [14] und [15] wurden zwar als relevant erachtet, allerdings lassen sie sich keinen spezifischen Maßnahmen zuordnen.

**Tab. 1:** Übersicht und Einordnung relevanter Publikationen nach Regionsbezug und Maßnahmen

Autor	Jahr	Regionsbezug		Maßnahmen (nach Auschra et al. [3])					
		Allgemein	Ländlich	Maßnahmen der Kontextgestaltung	Organisationsformen	Organisation	Technik	Profession	Mobilität
Ahrens [18]	2023	–	x	–	–	–	x	–	–
Auschra et al. [3]	2018	x	–	x	x	x	x	x	x
Beckers [19]	2015	x	–	–	–	–	x	–	–
Berghöfer et al. [9]	2020	–	x	x	x	–	x	–	x
Berndt et al. [20]	2019	–	x	–	–	–	x	–	–
Blank [16]	2019	–	x	x	–	–	–	x	–
Bohm et al. [21]	2022	–	x	x	–	–	–	–	–
Brinkrolf et al. [22]	2022	–	x	–	–	–	x	–	–
Geraedts et al. [13]	2022	–	x	–	–	–	–	–	–
Geuter et al. [23]	2016	–	x	x	–	–	–	–	–
Hämel et al. [24]	2017	–	x	–	–	–	–	–	x
Hildebrandt et al. [25]	2015	–	x	–	–	–	–	–	–
Müller et al. [26]	2018	x	–	x	–	–	–	–	–
Müller et al. [27]	2016	x	–	x	x	x	–	x	x
Niedersächsisches Ministerium für Soziales, Gesundheit und Gleichstellung [28]	2019	–	x	x	–	–	–	x	–
Schäfer et al. [17]	2015	–	x	x	–	–	–	–	–
Schmidt und Hintz [29]	2022	–	x	–	x	–	x	–	–
Schmiedhofer et al. [30]	2017	–	x	–	–	–	–	x	–
Schubert et al. [14]	2021	–	x	–	–	–	–	–	–
Schubert et al. [15]	2016	–	x	–	–	–	–	–	–
Schwartze und Wolf [31]	2017	–	x	–	–	–	–	–	x
Süss et al. [32]	2022	–	x	–	–	–	x	–	–
Weißenfeld [33]	2021	x	x	–	–	–	x	–	–

## Maßnahmen der Kontextgestaltung

Besonders im Fokus (Tab. 1) standen die Maßnahmen der Kontextgestaltung, wie beispielsweise die Niederlassungsförderung. So beschreibt und bewertet Blank das Versorgungsprojekt „Gute Ärzte braucht das Land“, das zwischen 2014 und 2017 durchgeführt wurde [16]. Untersucht wird, wie junge Ärzt:innen längerfristig für die Arbeit im ländlichen Raum gewonnen werden können. Als besonders wichtig haben sich organisatorische Rahmenbedingungen (v. a. zur Vereinbarkeit von Beruf und Familie) und die Entlastung von administrativen Aufgaben herausgestellt. Darüber hinaus sind die individuelle Förderung und Betreuung der jungen Ärzt:innen in einem generationenübergreifenden Team von Bedeutung.

Ein ähnliches Vorhaben beschreiben Schäfer et al. [17]. Das Projekt „Landpartie Fulda“, ebenfalls ein Projekt zur Förderung des hausärztlichen Nachwuchses im ländlichen Raum, wurde 2011 zur Förderung des allgemeinmedizinischen Nachwuchses auf dem Land ins Leben gerufen und von den Autor:innen evaluiert. Die Ergebnisse zeigen, dass sich am Ende eines Praktikums (2-wöchiges Blockpraktikum) von Medizinstudierenden in einer ländlichen Praxis die Motivation, später „hausärztlich ländlich“ tätig zu werden, signifikant steigerte. Das Vorhaben hat gezeigt, dass eine frühe Einbeziehung junger Studierender in die landärztliche Tätigkeit zusammen mit einer finanziellen Förderung die Attraktivität einer landärztlichen Tätigkeit erhöhen kann.

Für Kommunen stellt die Verbesserung der Gesundheitsversorgung (z. B. Anwerben von Ärzt:innen im Sinne einer Niederlassungsförderung) eine große Herausforderung dar, denn es mangelt oftmals an Unterstützung- und Beratungsangeboten. Geuter et al. beschreiben die Erfahrungen eines eingerichteten Kommunalbüros für ärztliche Versorgung, dass unter anderem Kommunen hinsichtlich der Verbesserung der ärztlichen Versorgung vor Ort berät [23]. Das Beratungsangebot wurde seitens der Kommunen stark nachgefragt, zentrales Thema war primär die Verbesserung der wohnortnahen hausärztlichen Versorgung. Die Evaluation bisher abgeschlossener Beratungsfälle zeigt, dass die empfohlenen Strategien aus kommunaler Sicht erfolgreich sein können. Empfohlen werden unter anderem eine Anbindung der Beratungsbüros an den öffentlichen Gesundheitsdienst sowie eine Vernetzung mit bestehenden Strukturen wie den Regionalen- oder Landesgesundheitskonferenzen.

Neben der Niederlassungsförderung ist das Thema Mobilität im Sinne von Maßnahmen zur Verbesserung der Versorgung im ländlichen Raum von Bedeutung. Mobile Einrichtungen zielen auf die Vermeidung regionaler Unterversorgung und adressieren oftmals vulnerable Bevölkerungsgruppen. Dabei können bestimmte Krankheitsbilder fokussiert oder eine umfassende, teils multiprofessionelle wohnortnahe Primärversorgung angestrebt werden [24]. Der Beitrag von Schwartze und Wolf thematisiert beispielhaft das Projekt „Rollende Arztpraxis“ im Landkreis Wolfenbüttel, ein Pilotprojekt, in dem eine rollende Arztpraxis (RAP) eingerichtet wurde, um eine wohnortnahe Gesundheitsversorgung speziell im Hinblick auf chronisch Kranke, multimorbide ältere Patient:innen zu gewährleisten [31]. Alles in allem wird ein positives Resümee des Projektes gezogen. Hinsichtlich der technischen Umsetzung ist die RAP erfolgreich gewesen. Aus Sicht der Patient:innen kann die Nachfrage nach wohnortnaher medizinischer Versorgung mit dem Leistungsspektrum eines normalen Hausarztes durch die RAP gedeckt werden. Besonders positiv hoben die Patient:innen die vergleichsweise langen Behandlungszeiten hervor, die zu einer hohen Zufriedenheit sowohl bei ihnen als auch bei den behandelnden Ärzt:innen geführt haben.

## Telemedizin und andere digitale Lösungen

Ahrens et al. beschreiben das Projekt „MeDiLand“ (Bestandteil des Projektes „Digitales Dorf“), in dem Maßnahmen erprobt und evaluiert wurden, die darauf abzielten, die medizinische Versorgung und das allgemeine Wohlbefinden in ländlichen Gebieten Bayerns durch den Einsatz digitaler Technologien zu verbessern [18]. Der Aufbau eines intersektoralen Telemedizinnetzwerks hatte das Ziel, die medizinische Versorgung in ländlich geprägten Regionen zu optimieren. Ein weiterer wichtiger Bestandteil war die Schaffung eines Dorfbusses mit digitaler Buchungsmöglichkeit als Ergänzung zum öffentlichen Nahverkehr, um die Mobilität zu verbessern und damit vor allem den Zugang zu medizinischen Dienstleistungen zu erleichtern. MeDiLand hat gezeigt, dass digitale Lösungen wie Telemedizin-Netzwerke und digitale Mobilitätsangebote das Potenzial haben, die medizinische Versorgung in diesen Gebieten erheblich zu verbessern. Das Projekt verdeutlicht jedoch auch gewisse Schwierigkeiten, insbesondere bei dem Bemühen, zusätzliche Unterstützung und finanzielle Mittel für eine weitergehende Umsetzung zu gewinnen.

2 Studien beschreiben die Anwendung teledermatologischer Ansätze in strukturschwachen und ländlichen Regionen – am Beispiel von Mecklenburg-Vorpommern [20, 34]. Berndt et al. zeigen, dass teledermatologische Ansätze in der Lage sind, die Versorgung in strukturschwachen oder ländlichen Gebieten zu verbessern, indem sie den Zugang zu spezialisierten dermatologischen Diensten erleichtern und die Versorgungseffizienz erhöhen. Bei Schuster et al. wurde jedoch auch deutlich, dass die Bereitschaft zur Nutzung von Teledermatologie in ländlichen Gebieten Bayerns noch gering ist, obwohl diese das Potenzial hat, die dermatologische Versorgung zu verbessen. Genannt werden Bedenken hinsichtlich fehlenden persönlichen Kontakts, der Qualität der medizinischen Leistung und des Datenschutzes.

Im Übersichtsbeitrag von Berghöfer et al. wurden Barrieren und förderliche Aspekte für die Entwicklung und Implementierung von Versorgungsmodellen im Allgemeinen untersucht sowie die Potenziale ihres Einsatzes identifiziert [9]. Aus den Ergebnissen lassen sich unter anderem folgende Thesen ableiten: 1) Die Tätigkeit von Ärzt:innen in Teilzeit und Angestelltenpositionen (u. a. mehr Bedeutung von Work-Life-Balance) wird zunehmen, 2) distanzüberbrückende telemedizinische Lösungen gewinnen an Bedeutung, 3) die stationäre Versorgung im ländlichen Raum könnte an Bedeutung gewinnen, 4) Managementkompetenzen für die Organisation und Kommunikation in komplexen Versorgungsmodellen müssen in den leistungserbringenden Professionen ausgebaut und mitfinanziert werden, 5) erfolgreich evaluierte Modelle müssen Aussicht auf Überführung in die Regelversorgung haben.

## Diskussion

Im Rahmen dieser Studie sollten Public-Health-Maßnahmen zur Sicherstellung der Versorgung in strukturschwachen und ländlichen Regionen identifiziert sowie existierende Evaluierungsergebnisse zum Effekt dieser Maßnahmen beschrieben werden. Die Untersuchung hat mehrere Aspekte verdeutlicht. Zunächst hat sich gezeigt, dass es mittlerweile eine Vielzahl verschiedenster konzeptioneller Ansätze und praktischer Maßnahmen zur Sicherstellung der Gesundheitsversorgung in ländlichen bzw. strukturschwachen Räumen gibt. Diese werden von Akteuren auf unterschiedlichen Ebenen umgesetzt, wie beispielsweise direkt durch die Kommunen (z. B. Vergünstigung von Bauplätzen für Ärzt:innen im Falle einer Niederlassung) oder durch Fördermaßnahmen (z. B. Niederlassungsförderung) auf Landesebene. Die Maßnahmen werden als Einzelmaßnahme [31] oder komplexes Bündel von Maßnahmen [21] umgesetzt, um die Versorgung einer unterversorgten Region sicherzustellen beziehungsweise zu verbessern.

Die Untersuchung hat aber auch verdeutlicht, dass nicht alle Maßnahmen gleichermaßen in die Praxis überführt werden, was vor dem Hintergrund der lokalen Strukturen, Gegebenheiten sowie Akteure einzelner Regionen durchaus nachvollziehbar ist. So hat sich gezeigt, dass Maßnahmen zur Verbesserung der Kontextgestaltung und neue Organisationsformen relativ häufig angewendet werden. Dazu zählen etwa die Förderung der Niederlassung von Ärzt:innen, die Unterstützung in der Ausbildung bzw. Weiterbildung von Ärzt:innen (AiW) sowie veränderte Organisationsformen des Praxisbetriebs. Im Hinblick auf die Niederlassungsförderung werden insbesondere finanzielle Anreize auf unterschiedlicher Ebene, wie beispielsweise vergünstigtes Bauland oder Förderung der Einrichtung einer Praxis, geboten. Stellt man diese Maßnahmen den Erwartungen beziehungsweise Wünschen angehender Ärzt:innen gegenüber [16, 35, 36], zeigt sich jedoch, dass es hier teilweise eine Diskrepanz zwischen Wunsch und Angebot gibt. Seitens junger Ärzt:innen werden beispielsweise lokale infrastrukturelle Gegebenheiten, wie Kindergärten, weiterführende Schulen, als wichtige Kriterien für eine Niederlassung angegeben, die jedoch seitens der Kommunen nicht unmittelbar umgesetzt werden können. So konnten Roick et al. [37] zeigen, dass finanzielle Anreize im Vergleich zu den Rahmenbedingungen für die Familie anscheinend keine allzu große Bedeutung haben. Dies verdeutlicht ein grundlegendes Problem bei der Niederlassungsförderung, nämlich dass zwar verschiedene Maßnahmen umgesetzt werden, diese, zumindest in Teilen, jedoch an den eigentlichen Wünschen der Ärzt:innen vorbeigehen. Darüber hinaus hat sich am Beispiel mobiler Gesundheitseinrichtungen auch gezeigt, dass Maßnahmen mangels Wirtschaftlichkeit wieder eingestellt werden mussten [31, 38].

Die Telemedizin nimmt eine gewisse Sonderstellung ein. Sie hat zwar keinen Einfluss auf die Niederlassungsförderung, jedoch ist sie im Kontext der Sicherstellung der Versorgung eine wichtige Säule für ländliche und strukturschwache Räume und kommt entsprechend häufig zu Anwendung. Telemedizinische Maßnahmen sind vor allem im Vergleich, beispielsweise zu Maßnahmen der Kontextgestaltung, in der Regel leicht umzusetzen und haben den Vorteil, dass die Bevölkerung in strukturschwachen Räumen einen Zugang zur Versorgung bekommt, ohne längere Wege auf sich nehmen zu müssen. Mittels telemedizinischer Maßnahmen können Diagnose- und Beratungsleistungen einfacher und schneller durchgeführt werden, Gleiches gilt für die Überwachung chronischer Erkrankungen oder die Früherkennung [32, 39, 40].

Angesichts der Vielzahl potenzieller und bereits durchgeführter Maßnahmen stellt sich die zentrale Frage nach deren Effekt sowie nach ihrer Implementierung in die Regelversorgung. Verglichen mit der insgesamt hohen Zahl und Vielfalt existierender Maßnahmen bzw. Initiativen werden nur sehr wenige evaluiert, sodass deren Effekt oder gar Nutzen kaum beurteilbar ist. Entsprechend wird eine verstärkte Evaluation von Maßnahmen gefordert [3, 9]. Dies ist auch deswegen nachvollziehbar, da Versorgungsmodelle nicht nur die Sicherstellung der Gesundheitsversorgung in ländlichen und strukturschwachen Regionen gewährleisten, sondern generell zur Qualitäts- und Effizienzsteigerung von Gesundheitsversorgung beitragen können.

Die Ursachen für die unzureichende Evaluation sind vermutlich darin zu suchen, dass Versorgungsmodelle selber sehr komplex sein können und sich damit eine Evaluation als entsprechend aufwendig gestaltet. Darüber hinaus fehlt es oftmals an konkreten Zielparametern, die über Zufriedenheit der Patient:innen, Erreichbarkeit und Versorgungsdichte hinaus auch medizinische Versorgungsqualität abbilden, wie zum Beispiel Morbidität oder Mortalität der lokalen Bevölkerung. Zudem stellt sich die Frage nach den Kriterien einer Evaluation. So kann ein Projekt, wie beispielsweise die „Rollende Arztpraxis“ [31], aus Sicht von Ärzt:innen und Patient:innen als Erfolg gewertet werden, am Ende der Pilotphase jedoch aufgrund einer defizitären ökonomischen Bewertung wieder eingestellt werden. Auch ist die Bewertung der Nachhaltigkeit von Maßnahmen schwierig. So haben beispielsweise Schäfer et al. [17] zwar im Rahmen des Projektes „Landpartie Fulda“ zeigen können, dass ein 2‑wöchiges Blockpraktikum von Medizinstudierenden in Lehrpraxen die Motivation, später „hausärztlich ländlich“ tätig zu werden, signifikant erhöht, jedoch unklar ist, ob dies seitens der Studierenden auch umgesetzt wurde. Dieses Beispiel zeigt auch, dass eine Evaluation über einen längeren Zeitraum erfolgen muss, was die Komplexität bzw. den Aufwand nochmals erhöht.

Im Vergleich zu den identifizierten Maßnahmen der Kontextgestaltung etc. wurden bzw. werden telemedizinische Maßnahmen hinsichtlich ihres Effektes tendenziell häufiger bewertet. Insgesamt spiegeln die identifizierten Projekte die dynamische Entwicklung und den zunehmenden Stellenwert telemedizinischer Ansätze in der modernen Gesundheitsversorgung in Deutschland wider. Die Ergebnisse belegen die Effektivität telemedizinischer Methoden in der Verbesserung der Zugänglichkeit und Qualität der medizinischen Versorgung, insbesondere in ländlichen und strukturschwachen Gebieten. Anzumerken ist jedoch, dass bislang nicht in allen strukturschwachen bzw. ländlichen Regionen eine ausreichende Infrastruktur (Breitbandversorgung) für eine adäquate und datenschutzkonforme telemedizinische Versorgung vorhanden ist und somit der Einsatz telemedizinischer Maßnahmen regional teilweise noch limitiert ist [41, 42].

Diese Studie unterliegt Limitationen. So kann keine Gewährleistung auf Vollständigkeit bei der Identifizierung aller relevanten Studien gegeben werden. Insbesondere die große Anzahl existierender Maßnahmen bzw. Versorgungsmodelle und deren Anwendung erscheint nach wie vor unübersichtlich, zumal davon auszugehen ist, dass viele lokale Maßnahmen (z. B. Einrichtung einer Beratungsstelle einer Kommune) und deren mögliche Evaluation möglicherweise nicht veröffentlicht wurden. Die teilweise komplexen Maßnahmen bzw. Versorgungsmodelle konnten im Rahmen dieser Studie zudem nicht im Detail bewertet werden. Das betrifft auch die Übertragbarkeit der Maßnahmen in die Regelversorgung sowie in andere Regionen. Anzumerken ist zudem, dass der Beitrag Maßnahmen zur Verhinderung von Krankheiten, der Verlängerung des Lebens und der Förderung von Gesundheit in strukturschwachen Regionen nicht berücksichtigt. Dies war zwar nicht Bestandteil der Untersuchung, sollte zukünftig jedoch mehr Beachtung finden.

## Fazit

Wenngleich es zahlreiche Maßnahmen bzw. Versorgungsmodelle zur Verbesserung der Gesundheitsversorgung in strukturschwachen Regionen gibt, ist deren Effekt oder gar Nutzen oftmals unklar. In der Regel ist dies auf eine mangelnde Evaluation zurückzuführen, die sich jedoch auch komplex und aufwendig gestaltet. Zudem mangelt es an Evaluationsmethoden und insbesondere an Kriterien für eine Evaluation. Es sollte auch eine Möglichkeit zur intensiveren Vernetzung von Akteur:innen aus Praxis geschaffen werden, um gemeinsam aus Misserfolgen zu lernen und Best-Practice-Beispiele auszutauschen. Insgesamt wird empfohlen, dem Thema Evaluation mehr Beachtung zu schenken und Methoden sowie Kriterien weiterzuentwickeln. Darüber hinaus sollte mit einer begleitenden Evaluation dafür gesorgt werden, dass Maßnahmen bei erfolgreicher Evaluierung bei Projektende nicht eingestellt, sondern bei entsprechendem Effekt in die Regelversorgung überführt werden. Alles in allem bleibt aber festzuhalten, dass die vorliegende Studie zahlreiche positive Ansätze zur Sicherstellung der Gesundheitsversorgung in strukturschwachen Regionen identifiziert hat, die es weiter zu verfolgen gilt.
